# Novobiocin, a Newly Found TRPV1 Inhibitor, Attenuates the Expression of TRPV1 in Rat Intestine and Intestinal Epithelial Cell Line IEC-6

**DOI:** 10.3389/fphar.2018.01171

**Published:** 2018-10-15

**Authors:** Qianying Liang, Xueli Lv, Qing Cai, Yun Cai, Boxin Zhao, Guofeng Li

**Affiliations:** ^1^Department of Pharmacy, Nanfang Hospital, Southern Medical University, Guangzhou, China; ^2^Rational Medication Evaluation and Drug Delivery Technology Lab, Nanfang Hospital, Southern Medical University, Guangzhou, China; ^3^General Hospital of Guangzhou Military Command of PLA, Guangzhou, China; ^4^Department of Pharmacy, the Third Affiliated Hospital of Guangzhou Medical University, Guangzhou, China; ^5^Guangdong Provincial Key Laboratory of New Drug Screening, Southern Medical University, Guangzhou, China

**Keywords:** novobiocin, capsaicin, TRPV1, inhibitor, expression

## Abstract

**Background and Purpose:** Novobiocin (NOVO), an ABC transporter inhibitor, decreases intestinal wall permeability of capsaicin (CAP), an ABC transporter substrate. However, the mechanism of this effect is not consistent with the action of NOVO as an ABC transporter inhibitor. We previously found that CAP can also be transported via TRPV1, which was site-specific in the permeability of CAP across the intestine. We explored the regulation by NOVO of TRPV1 in the present study.

**Methods:** Rats and transfected IEC-6 cells were used as the models to assess intestinal permeability and expression of TRPV1. Ussing chamber and intracellular accumulation were used to evaluate the influence of NOVO on the transport of CAP *in vitro*. The expression of TRPV1 was detected after administration of NOVO by qRT-PCR, western blot and immunofluorescent imaging. In addition, MTT and lactate dehydrogenase (LDH) were used to evaluate the cytotoxicity of NOVO in both rat and cell models. Finally, the effect of NOVO on the absorption of CAP *in vivo* was studied by LC-MS/MS.

**Results:** *In vitro* data showed that there existed a dose-dependent relationship in the range of concentration between 5 and 50 μM, and even 5 μM NOVO could decrease intestinal permeability of CAP across the intestine. Meanwhile, cytosolic accumulation of CAP decreased when NOVO was used simultaneously or 24 h in advance. NOVO exhibited an inhibition level similar to that of ruthenium red (RR) or SB-705498, a TRPV1-specific inhibitor. NOVO down-regulated TRPV1 expression in the intestine and in transfected cells in a concentration-dependent fashion, hinting that its inhibition of the permeability of CAP is due to its inhibition of TRPV1 expression. Immunofluorescent imaging data showed that the fluorescence intensity of TRPV1 was reduced after pre-treatment with NOVO and SB-705498. *In vivo* data further demonstrated that oral co-administration of NOVO decreased C_max_ and AUC of CAP in dosage-dependent ways, consistent with its role as a TRPV1 inhibitor.

**Conclusion:** NOVO could be a potential TRPV1 inhibitor by attenuating the expression of TRPV1 and may be used to attenuate permeability of TRPV1 substrates.

## Introduction

Transient receptor potential vanilloid member 1 (TRPV1) is a ligand-gated cation channel, which activates a series of cellular signals (Tominaga et al., 1998). Because TRPV1 is activated by CAP, TRPV1 has also been called the CAP receptor (Caterina et al., 1997). In addition to allowing influx of large cation such as FM1-43, tetraethylammonium (Meyers et al., 2003; Hellwig et al., 2004; Munns et al., 2015), TRPV1 has also been reported to allow permeability of cationic aminoglycoside antibiotics, including gentamicin (Ocana and Baeyens, 1991; Prado and Machado Filho, 2002). Inhibiting TRPV1 would attenuate the permeation of gentamicin across kidney epithelial and inner ear sensory cells. Hence it might protect against serious kidney and ear side effects induced by the administration of gentamicin (Myrdal and Steyger, 2005; Ishibashi et al., 2009). Furthermore, data provide evidence that gentamicin entered cell via cation channels and that this penetration was mediated by TRPV1 (Lee et al., 2013). Thus, TRPV1 may not only mediate the transport of cellular ions, but also constitute one of the transporting pathways for some drugs.

Our previous study revealed that the transmembrane permeability of CAP across the colon was remarkably higher than that across the other regions of the gastrointestinal mucous membranes of rats. This observation may be explained by the distribution of TRPV1 in the intestine, which has distinct differences across different parts of the intestine and was most expressed in the colon (Funakoshi et al., 2006). Moreover, colon-specific transport of CAP vanished in presence of RR, a non-specific antagonist of TRPV1. This observation suggested that TRPV1 is involved in transmembrane permeability of CAP (Duan et al., 2013). Coincidentally, our previous data showed that NOVO, a BCRP inhibitor, could also decrease permeability of CAP.

NOVO is an aminocoumarin natural antibiotic used to treat bacterial infections caused by staphylococci (such as methicillin-resistant *Staphylococcus aureus*), pneumococci, enterococci and many other Gram-positive organisms (Gombert and Aulicino, 1984; Jones, 1989; Schmutz et al., 2003). NOVO was demonstrated to have a specific inhibitory effect on BCRP and has been used with some antitumor drugs to block BCRP-mediated drug efflux, potentially reversing multidrug resistance (Su et al., 2007). ABC transporters, including “BCRP, P-gp and multidrug resistance protein 2 (MRP2),” were usually located in the serosa of intestine. ABC transporters significantly restrict oral absorption and efflux of substrates from the serosal side to the mucosal side of the intestinal wall (Sinko et al., 2004; Su et al., 2006; You et al., 2013). Therefore, ABC transporter inhibitors would increase the intracellular accumulation of drugs. However, NOVO did not exhibit these expected results. In fact, our data showed that NOVO decreased the absorption of CAP. These conflicting results suggested the inhibition of CAP permeability was not associated with BCRP. These findings hinted that there might exist other mechanisms which are not related to BCRP-mediated transport.

Some researchers have advised that some antibiotics should not be taken with spicy food (chili) because it might affect pharmacokinetics of these antibiotics. Sumano-Lopez et al. (2007) found the oral bioavailability of ciprofloxacin would increase by 70% when administrated in combination with 0.01–0.5% CAP. It was also reported that CAP affected the oral absorption rate of cephalexin and cefazolin. Importantly, the absorption rate returned to a normal level after co-administration of RR (Komori et al., 2007a,b), suggesting that the absorption of cephalexin and cefazolin was affected by intestinal TRPV1. Based on these findings, we speculated that the interaction between spicy foods containing CAP and these antibiotics may be induced by the TRPV1 pathway.

Until now, the mechanism by which NOVO decreases the transport of CAP across intestinal membranes has remained unclear. Considering CAP’s role as a TRPV1 substrate, we hypothesized that NOVO may play a novel role, as a TRPV1 inhibitor, and decrease the permeability of CAP. Therefore, in the present study, intestinal cells (IEC-6) and rats were chosen as test models. CAP was used as an indicator of TRPV1 transport, while RR (a non-specific TRPV1 inhibitor) and SB-705498 (a specific TRPV1 inhibitor) were used as positive controls to explore the influence of NOVO in regulating TRPV1 *in vitro* and *in vivo*.

## Materials and Methods

### Reagents and Materials

CAP (lot number: 048K5058V), HEPES (lot number: 016K54331), and Trizma base (lot number: 103K5411) were obtained from Sigma–Aldrich Chemical Co. (St. Louis, MO, United States). RR and NOVO were provided from Guangzhou Institute for Drug Control, China. HPLC-grade methanol and acetonitrile were purchased from Merck (Darmstach, Germany). SB-705498 was purchased from Skelleck Chemicals LCC (Houston, TX, United States).

### Animals

All of the animal experiments were performed according to the guideline of Experimental Animal Ethics Committee of Southern Medical University, and animal studies were reported in compliance with the ARRIVE guidelines (Kilkenny et al., 2010; McGrath and Lilley, 2015). We used 5–7-week-old male SD rats (200–250 g) which were purchased from the Laboratory Animal Center, Southern Medical University (Guangzhou, China; Permit number for rats: 44002100008791). Animal study was performed according to National Institutes of Health guide for the care and use of laboratory animals. The rats were fed with standard food and water and maintained on 12/12-h light/dark cycle in a temperature (20 ± 2°C) and were given 1 week to acclimate before experimental use.

### Ussing Chamber Experiments

The intestinal permeability of CAP *in vitro* was performed using Ussing chamber. For the *in vitro* permeability studies, CAP was prepared in 1% ethanol in oxygenated (O_2_/CO_2_, 95/5) HEPES buffer (3 M KCl, 1 M CaCl_2_, 1 M MgSO_4_, 8.18 g NaCl, pH 7.4), which was prepared daily, to yield final concentration of 100 μM. NOVO was also prepared in HEPES buffer to yield final concentration at 5, 10, 25, 50, 100, and 200 μM. Animal intestinal segments for the permeability study were prepared in accordance with the experimental method as described previously (Yodoya et al., 1994; Wallon et al., 2005; Duan et al., 2013). Briefly, male SD rats, weighting 240–260 g, were fasted for 18 h before each experiment and anesthetized by injecting 10 % chloral hydrate anesthesia (i.p.). Different portions of the rat intestine were excised and flushed with HEPES buffer, including jejunum (after the first 5 cm of the top of small intestine), ileum (the distal part of small intestine) and colon (proximal to cecal-colonic junction), and incubated in the ice-cold HEPES buffer. Next, 3–4 cm of the intestine was clipped, and the serosa was removed rapidly on an ice-cold glass. The intestinal segments were fixed in the Ussing chamber. Finally, 7 mL of HEPES buffer was added to the receiving side while an equal volume of drug solution to the dosing side. All the chambers were maintained at 37°C by using a warm water-circulating pump and a mixture of 95% O_2_ and 5% CO_2_ aerated to ensure the activity of the membrane. 0.5 mL of the sample was collected from the receiving side at 30, 60, 75, 90, and 120 min and a 0.5 mL aliquot of HEPES was added at the same side after each sampling point. All the samples were kept at -20°C till HPLC analysis.

### Preparation of Tissue Extract

Forty male SD rats (200–250 g) were used for orally administered experiment. The animals were arbitrarily distributed in eight different groups and each group was treated with its respective dose of calculated amount. Group I was orally administered 0.9% normal saline (5 mL⋅kg^-1^). Group II labeled as positive control was orally administered with 10 μM RR (5 mL⋅kg^-1^). Rats of Group III–VIII were treated with 5 mL⋅kg^-1^ of NOVO dissolved in 0.9% normal saline (5, 10, 25, and 50 μM, respectively). The animals were orally administered twice a day for 2 weeks. After another 14 days, animals were sacrificed and then the jejunum, ileum and colon tissue were excised. The intestinal tissues were frozen in liquid nitrogen, and then stored at -80°C for protein or ribonucleic acid (RNA) isolation.

### Cells Culture and Plasmid Transfection

The rat intestinal epithelial cell line IEC-6, purchased from Kunming Institute of Zoology. CAS, was cultured in Dulbecco’s Modified Eagle’s Medium (DMEM) (Gibco, Grand Island, NY, United States) supplemented with 10% fetal bovine serum (FBS; Gibco) at 37°C in a humidified atmosphere of 5% CO_2_.

The plasmid pENTER containing the TRPV1 coding sequence NM_080704 was used as template in a PCR with the following primers: 5′-CGCAAATGGGCGGTAGGCGTG-3′ (forward) and 5′-CCTCTACAAATGTGGTATGGC (reverse). And synthetic small interfering RNA (siRNA) specific for rat TRPV1 (TRPV1-siRNA) was purchased from Vigene Bioscience (Rockville, MD, United States) with the following sequences: 5′-AGCGCAUCUUCUACUUCAACU-3′ (forward) and 5′-UUGAAGUAGAAGAUGCGCUUG-3′ (reverse) for TRPV1-siRNA. The transfecting process was conducted by using the lipofectamin^®^ 3000 transfection reagent (Invitrogen^TM^, Carlsbad, CA, United States), according to the manufacturer’s protocol.

### Measurement of Cell Viability and Cytotoxicity

This experiment focused on evaluating the cyctoxicity of NOVO, CAP and RR in IEC-6/TRPV1^+^ and IEC-TRPV1^-^ cells and finding their dosing with non-cytotoxicity on the test cells separately. The MTT assay was used to identify non-toxic doses of NOVO, CAP and RR in IEC6/TRPV1^+^ and IEC6/TRPV1^-^ cells. The transfected cells were plated in 96-well plates. NOVO, CAP or RR was added to the wells, and the plates were incubated for 12 or 24 h. Optical density at 490 nm was used to evaluate cell viability in 96-well plates.

After determination of the non-cytotoxicity concentration, LDH Release Assay Kit (Nanjing Jiancheng Bioengineering Inc., Nanjing, China) was further used to check the influence of NOVO, CAP or RR at non-cytotoxic concentration on the plasma membrane of the transfected cells. The transfected cells were plated in 12-well plates. NOVO, CAP and RR at non-cytotoxic concentration from the MTT assay were added to wells after cells were plated, and the plates were incubated for 12 or 24 h. The cells were centrifuged at 1000 *g* for 5 min. Next, 50 μL of supernatants were aspirated and the rest of supernatants were discarded. The cells were washed twice with deionized water and then lysed by ultrasonic wave cracker. And then cell lysate and supernatants were analyzed by using LDH Release Assay Kit, according to the manufacturer’s instructions.

### CAP Accumulation Assay

The transfected cells were grown to 80% confluence in 12-well plates. The experiment was divided into three parts: (1) Cells were incubated with CAP (25 μM) alone, or in combination with SB-705498 (0.1 μM), or in combination with RR (10 μM), or in combination with NOVO (1, 5, and 10 μM), for 5, 15, 30, and 60 min; (2) Cells were incubated with CAP (25 μM) for 5, 15, 30, and 60 min at the beginning. SB-705498, RR or NOVO was then added to the medium for another 15, 30, and 60 min. (3) After pretreatment with SB-705498, RR or NOVO for 24 h, cells were incubated with CAP (25 μM) in culture medium at 37°C in 5% CO_2_ for 5, 15, 30, and 60 min, respectively. After incubation, the medium was removed, and cell layers were washed three times with ice-cold PBS and then extracted with Membrane Protein Extraction Kit (Thermo Fisher Scientific Inc, Rockford, IL, United States), according to the manufacturer’s protocol. The cytosolic protein extracts were stored at -80°C until LC-MS/MS analysis.

### Immunofluorescent Staining

The IEC-6/TRPV1^+^ cells were cultured on coverslips in 24-well plates overnight. Briefly, cells were stimulated with NOVO (10 μM), SB-705498 (0.1 μM) for 24 h. At the end of incubation, cells were fixed with 4% paraformaldehyde in 0.1 M PBS for 20 min. The cells were then treated by 0.25% (v/v) TritonX-100 for 5 min, and washed again. Followed by blocking in 5% (v/v) horse serum for 60 min, cells were then incubated with primary antibodies for the anti-TRPV1 receptor antibody (1:1000, NB100-1617, Novus) overnight in a 4°C refrigerator. Alexa Flour^®^ 488-conjugated goat anti-rabbit IgG (Zhongshan Goldenbridge Biotechnology) at 1:100 dilution was used as the secondary antibody that incubated cells for 60 min. Thereafter, the coverslips were mounted face down onto slides with mounting medium containing DAPI and stored at 4°C before microscopy. Fluorescence images were captured using a fluorescence microscope (Olympus, Japan).

### Quantitative Real Time (qRT)-PCR

qRT-PCR was performed to evaluate TRPV1 mRNA expression after NOVO treatment. The total RNA of the cells or tissues was isolated with an RNA isolation plus kit (Takara Shuzo, Kyoto, Japan), according to the manufacturer’s protocol. The quality of RNA was confirmed by agarose gel electrophoresis and an optical density measurement of A260/A280. The cDNAs were prepared using SYBR^®^ Premix Ex Taq^TM^II (Takara Shuzo, Kyoto, Japan) and the following primers: 5′-CACAGAGTGGACCCAGATAACG-3′ and 5′-CACTCGAGATAGACATGCCACC-3′ for TRPV1, and 5′-AGAAGGCTGGGGCTCATTTG-3′, 5′-AGGGGCCATCCACAGTCTTC-3′ for GAPDH were carried out by using the LightCycler480 System. The relative expression was calculated by the 2^-ΔΔC(t)^ method (Livak and Schmittgen, 2001).

### Protein Extract and Western Blot

After treatment, the cells or tissues were collected and washed with PBS. Membrane protein extracts were obtained with Membrane Protein Extraction Kit (Thermo Fisher Scientific Inc, United States), according to the manufacturer’s protocol. Protein concentrations were quantified by using the BCA Protein Assay kit (Beyotime, Shanghai, China). Equal amounts of protein were resolved by SDS-PAGE on a 10% polyacrylamide gel and transferred onto a polyvinylidene fluoride membrane. The membrane was incubated with antibodies against TRPV1 (Novus Biologicals, LLC. Catalog: NB100-1617) and GAPDH (Bioworld Technology, Inc., Catalog: BS6007M), followed by incubation with anti-rabbit secondary antibodies (Cell Signaling Technology, Inc., Catalog: 7074).

### *In vivo* Pharmacokinetics Study

SD rats, weighing 240–260 g, were fasted for 18 h before the study and orally administered CAP (10 mg⋅kg^-1^) in combination with different doses of NOVO (12.5 mg⋅kg^-1^, 25 mg⋅kg^-1^, 50 mg⋅kg^-1^, and 100 mg⋅kg^-1^) or 0.9% saline. Blood samples were collected into heparinized tubes before and at 30, 60, 120, 180, 210, 240, 270, 330, 390, and 720 min after administration. Blood samples were immediately centrifuged at 4000 *g* (4°C) for 10 min to obtain plasma. The plasma samples were stored at -80°C until LC-MS/MS analysis.

### Plasma Sample and Protein Sample Preparation

The plasma samples and protein sample with 100 μL were extracted with 400 μL of methanol involving IS (verapamil 50 ng⋅mL^-1^) by vortex-mix for 1 min, ultrasonic extraction for 1 h, and then centrifugation for 30 min at 15000 *g*. The supernatant was transferred into a borosilicate glass and 2 μL was subjected to LC-MS/MS analysis.

### Analytical System

#### HPLC

The concentrations of CAP and NOVO *in vitro* permeability studies were determined by HPLC system and the HPLC were respectively performed as described (Guo et al., 2014) and in Zuhowski et al. (1994). In brief, samples were injected into a C18 column (3.5 μm, 2.1 mm × 100 mm). An aliquot of 20 μL of sample solutions were injected. Elution solvents were waster- methanol (30–70, v/v) for CAP and water with 0.1% phosphoric acid-methanol (10–90, v/v) for NOVO. The wavelength were set at 280 nm (CAP) and 340 nm (NOVO), respectively.

#### LC-MS/MS

The LC-MS/MS system consisted of Agilent 6460 Triple Quadrupole LC/MS systems via an ESI interface. The samples were separated on an Agilent ZORBAX SB-C18 column (3.5 μm, 2.1 mm × 150 mm) and the column temperature was maintained at 30°C. The mobile phase consisted of acetonitrile and water contains 0.1% formic acid (70:30, v/v). The flow rate was 0.2 mL⋅min^-1^. The ion source was an electrospray ionization interface in the positive mode. The precursor/product ion transitions were monitored at m/z 306→137 for CAP and m/z 455→165 for verapamil. Other parameters were as follows: spray voltage, 4.0 kV; capillary temperature, 350°C; gas flow, 10 L⋅min^-1^.

### Statistical Analysis

The data and statistical analysis comply with the recommendations on experimental design and analysis in pharmacology (Curtis et al., 2015). Data in all experiments were presented as the mean ± SD. Significant differences were tested by one-way ANOVA, Student’s *t*-test and Fisher’s Least Significant Difference (LSD) test using SPSS 13.0 software. For all tests, *p* < 0.05 was considered as statistically significant.

## Results

### NOVO Exhibits Dose-Dependent Inhibition of Intestinal Permeability of CAP

RR is a non-specific antagonist of TRPV1 which significantly reduced permeability of CAP. The TRPV1 inhibition mediated by RR at 10 μM was significantly different in the ileum (1.46-fold) and colon (1.64-fold) when compared with the CAP-only control. Hence, 10 μM RR was used as the positive control in order to compare the effects of NOVO and RR on the transport of CAP across the intestine. As shown in **Table 1**, NOVO greatly inhibited the permeability of CAP across the intestine. 5–25 μM NOVO could significantly decrease the permeability across the jejunum, ileum and colon in a dose-dependent way. Meanwhile, 50 μM NOVO could also reduce the transport of CAP in the jejunum and colon. More interestingly, 50 μM NOVO displayed an effect similar to 10 μM RR on the transport of CAP. However, NOVO at lower doses, 10 and 25 μM exhibited a stronger inhibitory effect than did 10 μM RR.

**Table 1:** The contribution of NOVO and RR in the transport of CAP across the jejunum, ileum, and colon.

Compounds		Papp (×10^-6^, cm⋅s^-1^; fold-inhibition factor)		
		Jejunum	Ileum	Colon
CAP		3.14 ± 0.24 (1.00)	7.37 ± 0.99 (1.00)	19.97 ± 3.09 (1.00)
	+5 μM NOVO	1.96 ± 0.25^∗^ (1.60)	3.40 ± 0.76^∗^ (2.17)	8.28 ± 2.55^∗^ (2.41)
	+10 μM NOVO	1.36 ± 0.47^∗^ (2.31)	2.17 ± 0.58^∗^ (3.40)	6.05 ± 1.85^∗^ (3.30)
	+25 μM NOVO	1.60 ± 0.38^∗^ (1.97)	4.02 ± 0.42^∗^ (1.83)	5.65 ± 2.04^∗^ (3.54)
	+50 μM NOVO	2.11 ± 0.04^∗^ (1.49)	5.23 ± 0.25 (1.41)	9.79 ± 2.20^∗^ (2.04)
	+10 μM RR	2.54 ± 0.40 (1.23)	5.06 ± 0.57^∗^ (1.46)	12.18 ± 2.90^∗^ (1.64)

*Each compound was consisted of 100 μM CAP in the absence or presence of the indicated concentrations of NOVO or RR. Each value represents mean ± SD (n = 5). ^∗^p < 0.05, versus the group treated with CAP in the absence of NOVO or RR. The fold-inhibition factor of NOVO or RR (values given in parentheses) was calculated by dividing the Papp with CAP in the absence of NOVO or RR by that obtained in the presence of NOVO or RR.*

### The Contribution of TRPV1 Inhibitor in the Transport of NOVO

**FIGURE 1:**
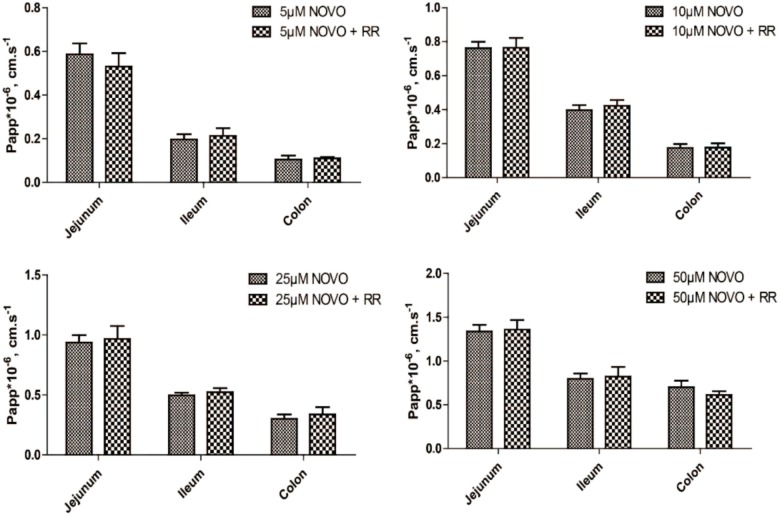
The effect of RR on the permeability of NOVO across the jejunum, ileum and colon. The samples contained the indicated concentrations of NOVO in the absence or presence of 10 μM RR. Each value represents mean ± SD (*n* = 5).

In order to determine whether the transport of NOVO across the intestine was related to TRPV1, we evaluated how RR affected the transport of NOVO. As shown in **Figure 1**, permeability of NOVO was much greater in the jejunum than in the ileum and colon. Additionally, there was no change in permeability of NOVO across these intestinal segments compared to the case when NOVO was administered in combination with RR.

### Characterization of TRPV1-Overexpressing (IEC-6/TRPV1^+^) and TRPV1-Knockdown (IEC-6/TRPV1^-^) Intestinal Epithelium Cell Lines 6

**FIGURE 2:**
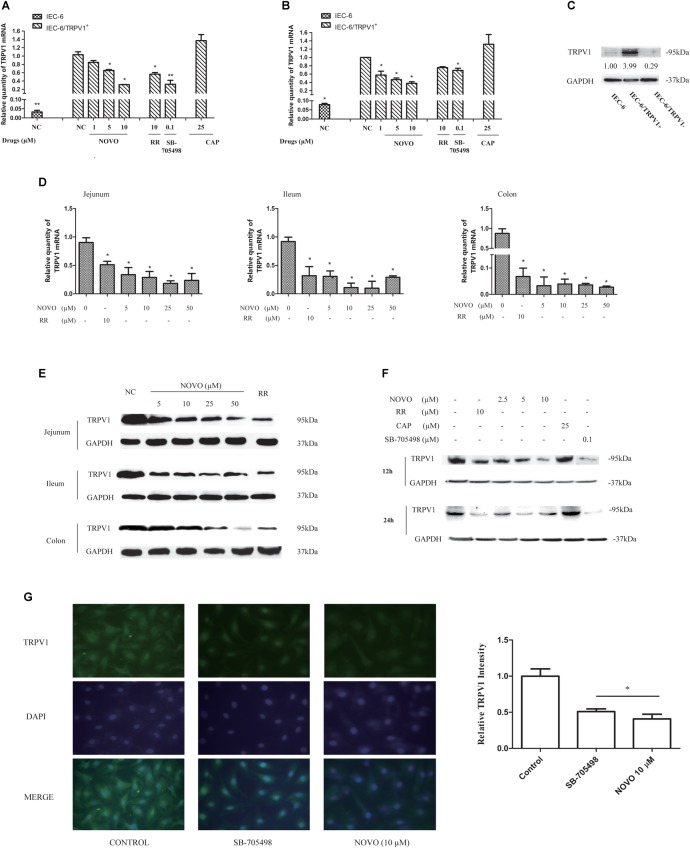
NOVO attenuated the expression of TRPV1. **(A,B)** Effects of NOVO on the expression of TRPV1 mRNA in IEC-6/TRPV1^+^ for 12 h **(A)** and 24 h **(B)**. IEC-6/TRPV1^+^ cells were treated with 2.5–10 μM NOVO for 12 or 24 h, or 10 μM RR for 12 or 24 h, or 0.1 μM SB-705498 for 12 or 24 h, 25 μM CAP for 12 or 24 h, or 0.1% DMSO for 12 or 24 h. Shown are the mean relative mRNA expression levels normalized to untreated cells from three independent experiments performed in triplicate with standard deviations. ^∗^*p* < 0.05 vs. control group. **(C)** The expression of TRPV1 protein in non-transfected and transfected cells. **(D)** Effects of NOVO on mRNA expression of TRPV1 in jejunum, ileum and colon. Shown are the mean relative mRNA expression levels normalized to NC from three independent experiments performed in triplicate with standard deviations. ^∗^*p* < 0.05, statistic versus NC. Each value represents mean ± SD (*n* = 5). Rats were exposed to different concentrations of NOVO (5, 10, 25, and 50 μM), RR (10 μM) or 0.9% saline (NC) through oral administration (p.o.) twice per day for 14 days. Total RNA was obtained from the intestinal tissues extracted from treated rats. **(E)** Effects of NOVO on the protein expression of TRPV1 in jejunum, ileum and colon. Total membrane proteins were obtained from the intestinal tissues extracted from treated rats. **(F)** Effects of NOVO on the expression of TRPV1 protein in IEC-6/TRPV1^+^. **(G)** Subcellular distribution of TRPV1 in the transfected cell lines. IEC-6/TRPV1^+^ cells were fixed for immunofluorescence testing followed by pre-treatment with NOVO (10 μM), SB-705498 (0.1 μM), or 0.1% DMSO for 24 h and then labeled with anti-TRPV1 antibody (green) and DAPI (blue). Original magnification × 400. ^∗^*p* < 0.05, statistic versus control. Each value represents mean ± SD (*n* = 5).

To elucidate the regulation by NOVO of TRPV1 activity, intestinal IEC-6 cells were transiently transfected with a plasmid containing the TRPV1 coding sequence. Successful overexpression (IEC-6/TRPV1^+^) and knockdown (IEC-6/TRPV1^-^) compared with untreated controls were verified by qRT-PCR (**Figures 2A,B**) and western blot (**Figure 2C**). Then we examined the individual effects of NOVO, CAP, RR, and SB-705498 on the survival of IEC-6/TRPV1^+^ and IEC-6/TRPV1^-^ cells by using a MTT assay. As shown in **Table 2**, more than 80% of the cells were viable up to a concentration of 10 μM NOVO, 25 μM CAP, and 10 μM RR for both IEC-6/TRPV1^+^ and IEC-6/TRPV1^-^ which had been incubated for 24 h with these inhibitors. It is known that SB-705498 has low cytotoxicity in transfected cells at doses ranging from 1 to 5000 nM. Hence, NOVO at concentrations of 2.5, 5, and 10 μM were used as low, medium, and high concentrations in assessing its mode of action. Similarly, 25 μM CAP, 10 μM RR, or 0.1 μM SB-705498 was used as the non-cytotoxic concentration in the experiments.

**Table 2:** Effects of NOVO, CAP, and the TRPV1 inhibitors on cell survival of the transfected cells.

Compounds	Concentration(μM)	Cell Survival			
		Incubation time (h)			
		12		24	
		IEC-6/TRPV1^+^	IEC-6/TRPV1^-^	IEC-6/TRPV1^+^	IEC-6/TRPV1^-^
NOVO	50	1.065 ± 0.125	1.123 ± 0.293	0.668 ± 0.124	0.591 ± 0.261
	40	1.018 ± 0.091	1.051 ± 0.214	0.752 ± 0.291	0.647 ± 0.015
	20	1.113 ± 0.102	1.011 ± 0.194	0.744 ± 0.232	0.749 ± 0.138
	10	1.156 ± 0.135	1.101 ± 0.101	0.954 ± 0.012	0.861 ± 0.088
	5	1.118 ± 0.201	1.128 ± 0.274	1.282 ± 0.203	0.952 ± 0.134
	2.5	1.139 ± 0.157	1.063 ± 0.361	1.326 ± 0.252	1.113 ± 0.177
	1.25	1.208 ± 0.172	1.261 ± 0.512	1.353 ± 0.192	1.192 ± 0.099
CAP	50	0.820 ± 0.201	0.792 ± 0.316	0.739 ± 0.012	0.801 ± 0.171
	25	0.896 ± 0.199	0.851 ± 0.216	0.822 ± 0.102	0.878 ± 0.096
	12.5	1.029 ± 0.180	0.969 ± 0.310	0.916 ± 0.163	0.934 ± 0.111
RR	20	0.886 ± 0.319	0.776 ± 0.409	0.577 ± 0.153	0.648 ± 0.119
	10	1.023 ± 0.252	0.853 ± 0.128	0.822 ± 0.024	0.834 ± 0.032
	5	1.102 ± 0.102	0.972 ± 0.201	0.962 ± 0.062	0.902 ± 0.100
SB-705498	0.001	1.114 ± 0.190	1.261 ± 0.268	1.018 ± 0.061	0.999 ± 0.082
	0.01	1.101 ± 0.236	1.158 ± 0.144	1.129 ± 0.185	0.968 ± 0.218
	0.1	1.274 ± 0.223	1.169 ± 0.333	1.118 ± 0.139	0.962 ± 0.184
	1	1.203 ± 0.139	1.001 ± 0.229	1.052 ± 0.100	0.933 ± 0.077
	5	1.276 ± 0.156	0.931 ± 0.296	0.976 ± 0.098	0.907 ± 0.039

*Cell survival was determined by MTT assays as described in Section “Materials and Methods.” Data are the mean ± SD of at least three independent experiments performed in triplicate. Survival rate (%) = [1- (OD treated – OD blank)/(OD control – OD blank)] × 100%; OD, optical density. Cell survival > 80% means non-cytotoxicity to cells.*

Analysis of cell membrane integrity was carried out using the LDH release assay. This assay was used to re-evaluate whether there was any damage to the cell membranes in the presence of NOVO, CAP, RR, and SB-705498 at the non-cytotoxic concentrations determined by the MTT assay. As shown in **Table 3**, there was not any significant difference in LDH release after cells were treated with NOVO (2.5, 5, and 10 μM), CAP (25 μM), RR (10 μM), and SB-705498 (0.1 μM) for 12 and 24 h, compared with the control. This meant no damage was induced by NOVO, CAP, RR, and SB-705498.

**Table 3:** Effects of NOVO, CAP, and the TRPV1 inhibitors on cell membrane integrity of IEC-6/TRPV1^+^ cells (percentage of LDH release was used as an index).

Drugs	Conc.(μM)	LDH release (gprot⋅mL ^-1^)			
		12 h		24 h	
		IEC-6/TRPV1 ^+^	IEC-6/TRPV1^-^	IEC-6/TRPV1 ^+^	IEC-6/TRPV1^-^
Control		0.593 ± 0.086	0.668 ± 0.071	0.487 ± 0.073	0.572 ± 0.063
RR	10	0.406 ± 0.056	0.552 ± 0.039	0.301 ± 0.061	0.501 ± 0.094
SB-705498	0.1	0.469 ± 0.124	0.509 ± 0.155	0.423 ± 0.149	0.537 ± 0.019
CAP	25	0.380 ± 0.165	0.680 ± 0.205	0.311 ± 0.051	0.462 ± 0.101
NOVO	1	0.415 ± 0.169	0.638 ± 0.131	0.353 ± 0.026	0.433 ± 0.127
	5	0.356 ± 0.090	0.651 ± 0.120	0.300 ± 0.052	0.554 ± 0.103
	10	0.337 ± 0.005	0.535 ± 0.119	0.299 ± 0.072	0.502 ± 0.106

*Cell cytotoxicity was determined by a LDH release assay kit as described in Section “Materials and Methods.” All data are the mean ± SD values of the ratio of release of LDH into cell culture supernatants to intracellular fluid. Cells treated with 1% ethyl alcohol in 10% culture medium were considered as the control group. LDH activity (U⋅mL^-1^) = [(OD treat – OD control)/(OD standard – OD blank)] × 0.2 × N. LDH activity of Suspension (U⋅gprot^-1s^) = LDH activity/protein concentration; N, dilution ratio; 0.2, the concentration of standard solution. LDH release % (gprot⋅mL^-1^) = LDH activity of supernatants/LDH activity of Suspension.*

### NOVO Attenuated the Expression of TRPV1 in the Intestine

qRT-PCR and western blot analysis were performed to detect any change in mRNA and protein levels of TRPV1 upon pretreatment with NOVO, respectively. **Figure 2D** shows the influence of NOVO on the expression of TRPV1 mRNA in the rat intestine after 14 days of p.o. administration of NOVO. The data demonstrate that the TRPV1 mRNA level decreased markedly in a NOVO-dose-dependent manner. The mRNA expression analysis showed less mRNA in the jejunum than in the ileum, and less mRNA in the ileum than in the colon. Similarly, NOVO also decreased the TRPV1 protein level in a dose-dependent manner (**Figure 2E**). However, 5 μM NOVO was not sufficient to attenuate TRPV1 protein expression.

### NOVO Down-Regulated TRPV1 mRNA and Protein Expression in IEC-6/TRPV1^+^ Cells

NOVO could also dramatically attenuate the expression of TRPV1 mRNA (**Figures 2A,B**) and protein (**Figure 2F**) in a concentration-dependent manner over 24 h in IEC-6/TRPV1^+^ cells. Moreover, NOVO displayed a similar suppression effect on the expression of TRPV1 as did RR and SB-705498.

To further validate this change in TRPV1 expression, we investigated the subcellular localization of TRPV1 in IEC-6/TRPV1^+^ cells (**Figure 2G**). Immunofluorescent imaging data showed that TRPV1 was distributed diffusely throughout the cell with detectable cell surface expression in the untreated group, which corresponded with the protein expression levels identified with western blot analysis. The fluorescence intensity of TRPV1 was reduced after pre-treatment with NOVO (10 μM) and SB-705498.

### NOVO Inhibited the Accumulation of CAP in Cells

**FIGURE 3:**
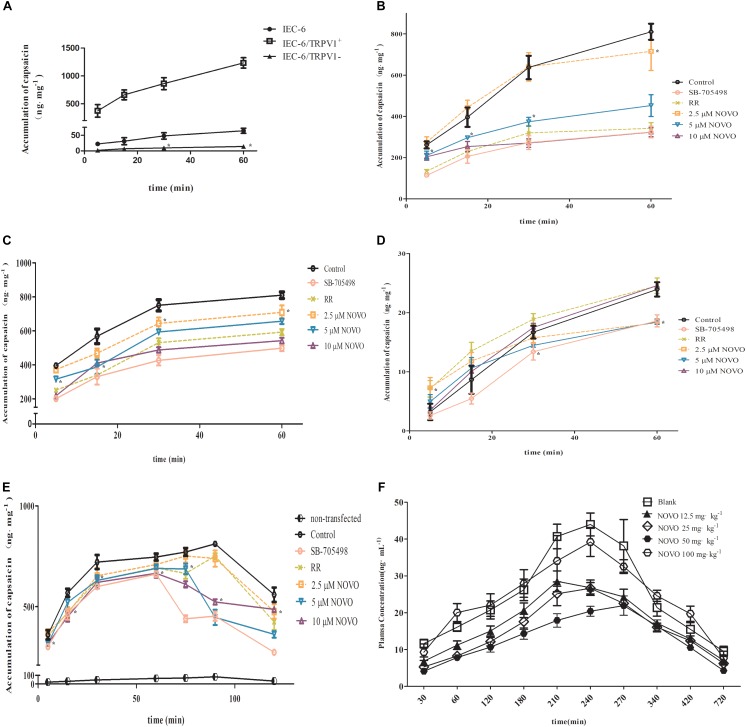
Effects of NOVO on the intracellular accumulation of CAP in cells and on the pharmacokinetics of CAP *in vivo*. **(A)** The accumulation of CAP in non-transfected and transfected cells. Cells were treated with 25 μM CAP for 5, 15, 30, and 60 min. ^∗^*p* < 0.05, statistic versus control. **(B)** The accumulation of CAP after different pre-treatments in TRPV1-overexpressing IEC-6 cells. Pre-treatment (24 h) of IEC-6/TRPV1^+^ with 2.5–10 μM NOVO, RR (non-specific inhibitor), SB-705498 (specific inhibitor) or 0.1% DMSO. Subsequently, IEC-6/TRPV1^+^ cells were incubated with 25 μM CAP for 5, 15, 30, and 60 min. ^∗^*p* < 0.05, statistic versus control. **(C)** The accumulation of CAP after the administration of CAP in combination with 2.5–10 μM NOVO, RR (non-specific inhibitor), SB-705498 (specific inhibitor) or 0.1% dimethyl sulfoxide (DMSO) in TRPV1-overexpressing IEC-6 cells, respectively. ^∗^*p* < 0.05, statistic versus control. **(D)** The accumulation of CAP after the administration of CAP in combination with 2.5–10 μM NOVO, RR (non-specific inhibitor), SB-705498 (specific inhibitor) or 0.1% DMSO in TRPV1-knockdown IEC-6 cells. ^∗^*p* < 0.05, statistic versus control. **(E)** The effect of NOVO, RR and SB-705498 on the accumulation of CAP in IEC-6/TRPV1^+^. Cells were treated with 25 μM CAP and then treated with NOVO, RR, or SB-705498 at 60 min. ^∗^*p* < 0.05, statistic versus control. **(F)** Mean plasma concentration versus time profiles of CAP after the oral administration of CAP (10 mg⋅kg^-1^) in combination with NOVO (12.5–100 mg⋅kg^-1^) to rats. *In vivo* data are represented as the mean ± SD values from six independent experiments. Cell data were represented as the mean ± SD values from three independent experiments performed in triplicate.

To validate the relationship between TRPV1 expression and permeability of CAP, the intracellular accumulation of CAP through cell membranes was examined. We found first that the accumulation of CAP in IEC-6/TRPV1^+^ cells surpassed that in IEC-6 cells. However, IEC-6 cells with TRPV1 knockdown significantly decreased the accumulation of CAP (**Figure 3A**). Second, IEC-6/TRPV1^+^ cells were exposed to 2.5–10 μM NOVO, 10 μM RR, or 0.1 μM SB-705498 for 24 h prior to incubation with CAP for 60 min. **Figure 3B** shows that NOVO concentrations (2.5–10 μM) exhibited a positive correlation with a decrease in the accumulation of CAP in the cells. Additionally, 10 μM NOVO displayed similar effects as SB-705498 and RR did in the transport of CAP. Third, after successful construction, transfected cells were exposed to CAP in the presence of NOVO, RR or SB-705498. Fourth, transfected cells were exposed to 2.5–10 μM NOVO, 10 μM RR, or 0.1 μM SB-705498 for 24 h in the presence of CAP for 60 min. Compared to the control, NOVO exhibited strong inhibition of the accumulation of CAP in IEC-6/TRPV1^+^ cells (**Figure 3C**). Furthermore, 10 μM NOVO displayed a similar effect as SB-705498 did in the transport of CAP. However, in IEC-6/TRPV1^-^, the intracellular accumulation of CAP was far less than that in IEC-6. Moreover, the accumulation of CAP in IEC-6/TRPV1^-^ did not change when the cells were treated with RR, SB-705498, or NOVO (**Figure 3D**). Finally, IEC-6/TRPV1^+^ cells were exposed to CAP alone for 60 min prior to administration of NOVO, RR, or SB-705498. The plot indicates that the accumulation of CAP decreased immediately at 75 min after cells were treated with SB-705498 and NOVO (5 and 10 μM) (**Figure 3E**). SB-705498 and 10 μM NOVO exhibited sustained inhibition of the accumulation of CAP. Compared to the control, NOVO at 2.5 and 5 μM decreased the permeation of CAP.

### NOVO Decreased the Absorption of CAP After Oral Co-administration

Plasma samples for analysis of CAP after oral administration of different doses of NOVO were taken from anesthetized rats in the *in vivo* study. The peak time (T_max_) of CAP was about 240 min and it did not change with dosage. C_max_ and AUC_0-720min_ were gradually attenuated as the dose of NOVO increased in the 12.5-50 mg⋅kg^-1^ range (**Table 4**), indicating that NOVO could inhibit the absorption and affect the pharmacokinetic properties of CAP. However, 100 mg⋅kg^-1^ NOVO had no impact on the pharmacokinetic properties of CAP compared with the CAP-only group. As shown in **Figure 3F**, the variation of plasma concentration versus time profiles of CAP was related to the dose of NOVO after oral administration of NOVO (12.5-50 mg⋅kg^-1^).

**Table 4:** Pharmacokinetic parameters of CAP after the oral administration of CAP (10 mg⋅kg^-1^) in combination with NOVO (12.5–100 mg⋅kg^-1^) in rats.

Dosing Group	N	C_max_ (ng⋅mL^-1^)	T_max_ (min)	AUC_0-720__min_(ng⋅min⋅mL^-1^)
Blank	5	52.157 ± 8.920	234 ± 25.100	13793.660 ± 1862.059
NOVO 12.5 mg⋅kg^-1^	5	32.534 ± 2.248	222 ± 26.832	10203.630 ± 432.204
NOVO 25 mg⋅kg^-1^	5	29.874 ± 3.355^∗^	234 ± 25.100	9217.946 ± 326.929^∗^
NOVO 50 mg⋅kg^-1^	5	25.082 ± 0.957^∗^	264 ± 13.416	7874.992 ± 629.840^∗^
NOVO 100 mg⋅kg^-1^	5	43.137 ± 8.829	224 ± 25.100	14547.74 ± 1084.295

*All data are represented as the mean ± SD values from six independent experiments. ^∗^p < 0.05, statistic versus Blank.*

## Discussion

Few studies have shown how CAP permeates across the membranes. In theory, CAP is a lipophilic compound (Georgescu et al., 2017) and its logP is 4 (Iida et al., 2003), which means it might permeate lipid bilayers. Several groups have proposed CAP may interact with lipids within cell membranes. Hanson et al. indicated that CAP could travel through lipid bilayers by interacting with TRPV1 in molecular dynamics simulations (Hanson et al., 2015). However, most drugs/compounds do not have only way to access the cytoplasm. Many parameters, including pKa and solubility of drugs/compounds, can also play a role in determination of their transport pathways. Furthermore, our previous study demonstrated that the highest permeation of CAP was colon-specific and therefore could be a consequence of the colon-specific distribution of TRPV1 (Duan et al., 2013). In addition, some researchers indicated that TRPV1 could be a transport pathway to regulate the absorption of certain drugs, including CAP (Myrdal and Steyger, 2005; Duan et al., 2013; Lee et al., 2013). Therefore, we hypothesized that CAP may not only be an agonist of TRPV1 (Vriens et al., 2009), but also a transporter substrate of TRPV1.

We used the intestinal cell line IEC-6 to explore whether CAP permeates intestinal cells via TRPV1. IEC-6 is a common model for *in vitro* experiments and has characteristics similar to intestinal basement membrane. These cells have microvilli, centrioles, Golgi complexes, and junctional complexes according to an ultrastructural study (Quaroni et al., 1979). Due to the characteristics of IEC-6 cells, this cell line has also been used in studies of hepatotoxicity (Zhou et al., 2017), tight junctions (Yu et al., 2016) and TRP channels (Kono et al., 2013; Yamawaki et al., 2014). Cells were transiently transfected with plasmid to produce TRPV1- overexpressing and TRPV1-knockdown cells. The data showed that knockdown significantly suppressed the permeability of CAP in IEC-6/TRPV1^-^ compared to non-transfected cells. Moreover, the permeability of CAP was greatly increased in IEC-6/TRPV1^+^. The above results indicated that overexpression of TRPV1 could accelerate the permeation of CAP. TRPV1 knockdown suppressed the intracellular accumulation of CAP. Together, these findings suggested that TRPV1 plays an important role in CAP transport through membranes as we had hypothesized.

Since we had demonstrated that TRPV1 affected CAP traveling through membranes, we used rat intestinal tissues to evaluate the effect of NOVO on the permeability of CAP across the intestine with an Ussing chamber system. First, intestinal samples were treated with concentrations of NOVO ranging from 5 to 50 μM to evaluate the contribution of NOVO to the transport of CAP *in vitro*. The data showed that NOVO at 5–25 μM significantly decreased the permeability of CAP, in a concentration-dependent manner. RR, a non-specific blocker of TRPV1, interferes with the opening of TRPV1 channels, thereby decreasing calcium entry and subsequent peptide release (Dray et al., 1990; Lou et al., 1991). A previous study showed that RR produced long-lasting, selective, and reversible inhibition of CAP-induced plasma extravasation in rat tracheas (Brokaw and White, 1995). Loris et al. found that pretreatment with RR of the ileum abolished the response to CAP and blocked TRPV1 activation (Chahl, 1989). Therefore, we used RR (10 μM) as a positive control in order to compare the effect of NOVO and RR in the transport of CAP across the intestine. **Table 1** shows that RR decreased the permeability of CAP across the jejunum, ileum, and colon. Furthermore, NOVO showed an even stronger (5–25 μM) effect on inhibiting permeability than RR did. However, this led to a question: was the inhibitory contribution of NOVO a result of its role as a TRPV1 inhibitor or a result of its role as a competitive substrate? As shown in **Figure 1**, RR did not affect the transport of NOVO. In addition, we had found that the TRPV1-mediated permeability of CAP across the colonic mucosa was remarkably higher than that across the jejunal or ileac mucosa. NOVO itself did not show transport permeability similar to that of CAP, suggesting that NOVO was not a competitive substrate of TRPV1. This excluded the possibility that NOVO decreased intestinal permeability of CAP through its competitive binding to the binding site of TRPV1. Preliminary results suggested NOVO might inhibit the transport function of TRPV1.

RR is a non-specific antagonist of TRPV1. RR inhibits a large number of TRP channels, ryanodine receptors and many other membrane proteins (Gustafsson et al., 2005; Maqboul and Elsadek, 2018). In addition, RR is used as a cell surface stain in histology suggesting that it may interact with cell membranes. Therefore, we chose a specific TRPV1 antagonist, SB-705498 (Gunthorpe et al., 2007; Bareille et al., 2013; Holland et al., 2014), as another positive control in our intestinal cell models in order to compare the effect of the inhibition of TRPV1 and the accumulation of CAP in the presence or absence of NOVO.

Next, we used the TRPV1-overexpression and TRPV1-knockdown cell models to evaluate how NOVO affected cell permeability of CAP with different levels of expression of TRPV1. Due to the apparent binding affinity of CAP to TRPV1, the accumulation of CAP may increase in the presence of membrane protein. Furthermore, the protein binding ratio of CAP is higher than 90% (data not shown). Hence, the cells were separated into cytosolic and membrane fractions. The concentrations of CAP in cytosolic fractions were used to estimate intracellular accumulation. These samples excluded the membrane proteins, including TRPV1, to which CAP may bind in the membrane. First, we explored the intracellular accumulation of CAP in the presence of NOVO or SB-705498 in IEC-6/TRPV1^+^. The data showed that both NOVO and SB-705498 decreased the accumulation of CAP in the cells. Second, we evaluated the effect of SB-705498 and NOVO on the intracellular accumulation of CAP in IEC-6/TRPV1^-^. The data showed that the accumulation of CAP did not change in IEC-6/TRPV1^-^ cells in the presence of NOVO or SB-705498. These results indicated that TRPV1 played a key role in the effect of NOVO on the accumulation of CAP. Third, IEC-6/TRPV1^+^ cells were exposed to CAP alone for 60 min, and then NOVO or SB-705498 was added in the culture medium at 60 min. The accumulation of CAP decreased immediately after cells were exposed to SB-705498 or NOVO. Both 5 and 10 μM NOVO showed similar inhibition as SB-705498 did in the accumulation of CAP. These data demonstrated that the increasing accumulation of CAP in the TRPV1-overexpressing cells would decrease rapidly if followed with the specific TRPV1 inhibitor. This observation verified the effect of NOVO on the TRPV1 pathway.

After verifying the effect of NOVO on cellular permeability of CAP, we tried to explore the effects of NOVO on the expression of TRPV1. Rats and TRPV1-overexpressing cells were used as a model to evaluate any correlation between NOVO treatment and TRPV1 expression. TRP channels are integral membrane proteins and require a lipid membrane environment for proper function (Huynh et al., 2014). Several studies have determined the structures of the TRPV1 and revealed its mechanisms of ligand and lipid action (Cao et al., 2013; Liao et al., 2013; Gao et al., 2016), which verified TRPV1 stable expression in the cell membrane (Melnick and Kaviany, 2018). And Salvador et al. indicated that TRPV1 was located on the cell surface in both primary DRG neurons and transfected HEK293 (Sanz-Salvador et al., 2012). Photomicrographs shown in **Figure 2G** also illustrate that TRPV1 was distributed throughout the transfected cells. Moreover, both the specific TRPV1 inhibitor and NOVO could reduce the fluorescence intensity of TRPV1, compared with the untreated control. TRPV1, known as integral membrane protein, might exhibit the effect as a transporter when it located on the cell surface. Hence, we used Membrane Protein Extraction Kit for enrichment of integral membrane protein for better analysis. The results showed that increasing the concentration of NOVO dose-dependently suppressed the expression of TRPV1 mRNA and protein. It further suggested that NOVO might inhibit TRPV1 by affecting its expression in the intestine and inhibiting the permeation of CAP through membrane. Based on these findings, IEC-6/TRPV1^+^ cells were pre-incubated with RR, SB-705498, and NOVO for 24 h in order to evaluate any effects of these changes on CAP permeation. The results indicated that NOVO exhibited similar effects as the TRPV1 specific inhibitor SB-705498 in inhibiting the accumulation of CAP after pre-incubation for 24 h. Thus, the reason why the permeability of CAP decreased was that NOVO inhibited the expression of TRPV1. It is known that NOVO is a DNA gyrase inhibitor, and it has been shown that NOVO can affect the regulation of the transcription and translation of many genes (Gmuender et al., 2001; Chatt et al., 2014; Byrd et al., 2016). Therefore, decreased TRPV1 expression may be regulated by one of the genes that are affected by NOVO. However, it is unclear how the signaling pathway works, and more studies should be undertaken.

On the basis of the *in vitro* study, LC-MS/MS was used to detect the plasma concentration of CAP after oral administration of NOVO and to verify the impact of NOVO on the absorption of CAP *in vivo*. The *in vivo* pharmacokinetic study found that NOVO affected the pharmacokinetic properties of CAP in a dose-dependent fashion. This study showed that 12.5–50 mg⋅kg^-1^ NOVO significantly attenuated C_max_ and AUC_0-720__min_ of CAP in a dosage-dependent manner. However, NOVO at 100 mg⋅kg^-1^ did not affect C_max_ and AUC_0-720__min_ of CAP when compared with the CAP-alone group. In addition, we did find that 50 μM NOVO did not affect the permeability of CAP (**Table 1**). We found that the permeability of CAP increased when the concentration was higher than 100 μM in the Ussing chamber experiment (data not shown). Based on these results, we hypothesized that NOVO would inhibit TRPV1 only at certain dosage. Once the dose of NOVO was out of range, such as the 100 mg⋅kg^-1^ in our *in vivo* experiment, the weakening inhibition may be due to saturation or some other unknown effects that lead to an enhancement of the permeation of CAP across the intestine. It is well known that TRPV1 can also affect TJ and regulate the expression of TJ -related proteins, such as occludin, ZO-1, and ZO-2 (Li et al., 2015). In addition, the paracellular pathway, an important pathway for intercellular permeation, is mainly restricted by TJ located on the apical side of epithelial cells (Gunzel and Fromm, 2012). The controlled opening of TJ is a way to increase the absorption of drugs across the epithelium. Hence there might be a possibility, based on the above results, that high concentrations of NOVO would increase the opening of TJ in the intestine, and even damage the structure of TJ, which may have resulted in increasing the permeation of CAP across the intestine. Further investigations are yet to be done to verify this hypothesis. Additional work should also be done using fluorescent CAP analogs (Li et al., 2011) to examine the intracellular accumulation of CAP and the effect of NOVO on CAP internalization (Sanz-Salvador et al., 2012), which would define whether the above findings are specific to CAP or can be generalized to all TRPV1 substrates.

In this study, we demonstrate for the first time that NOVO can regulate the expression of TRPV1 and that this regulation is concentration-dependent. Moreover, we have shown that inhibition of TRPV1 expression might affect the transport of CAP across the intestine and its accumulation in cells. As far as human tissues are concerned, altered TRPV1 expression in nerve fibers has been demonstrated in biopsies from patients with rectal hypersensitivity or inflammatory bowel disease (IBD). Moreover, TRPV1 expression correlated with the degree of abdominal pain in IBD (Yiangou et al., 2001; Chan et al., 2003; Akbar et al., 2008). In rats, stress-induced visceral hypersensitivity, which is dependent on mast cell degranulation and subsequent TRPV1 activation, occurs in the absence of overt inflammation (Kihara et al., 2003; Miranda et al., 2007; van den Wijngaard et al., 2009). NOVO has been clinically used for decades and the therapeutic dose in human is 2 g per day (Kennedy et al., 1995). NOVO can exert its antibiotic effects and at the same time have a significant inhibitory effect on TRPV1 at equivalent doses in rats. The potency of NOVO suggests that it might be a useful therapeutic agent for rectal hypersensitivity or IBD due to its inhibition of TRPV1 along with its antimicrobial actions. It will be interesting to explore whether the inhibition of TRPV1 is specific to aminocoumarin antibiotics or other groups of antibiotics sharing the same properties.

## Conclusion

This study demonstrates that NOVO may be a potential TRPV1 inhibitor. In our knowledge, this study is the first of its kind to report that NOVO attenuates the expression of TRPV1 in cells and tissues *in vitro* and *in vivo*. Therefore, NOVO can be used to inhibit the transport of TRPV1 substrates and regulate TRPV1 expression.

## Author Contributions

QL, GL, and BZ designed the experiment and interpreted the results. QL and QC analyzed the data and wrote the manuscript. QL, XL, and YC performed the experiments. All the authors approved the final manuscript.

## Conflict of Interest Statement

The authors declare that the research was conducted in the absence of any commercial or financial relationships that could be construed as a potential conflict of interest.

## Abbreviations

ABC: ATP-binding cassette protein family
AUC: Area Under Curve
BCRP: breast cancer resistant protein
CAP: capsaicin
C_max_: maximum plasma concentration
h: hour
IEC-6/TRPV1^+^: overexpression-TRPV1 IEC-6 cells
IEC-6/TRPV1^-^: TRPV1-knockdown IEC-6 cells
LDH: lactate dehydrogenase
logP: octanol/water partition coefficients
min: minute
MTT: 3-(4,5-dimethyl-2-thiazolyl)-2,5-diphenyl-2-H-tetrazolium bromide
NOVO: novobiocin
Papp: Apparent Permeability
qRT-PCR: quantitative real time PCR
RR: ruthenium red
SD: Sprague Dawley
TJ: tight junction
T_max_: time to peak
TRPV1: Transient receptor potential cation channel subfamily V member 1
